# Internet Gaming Disorder: Exploring Its Impact on Satisfaction in Life in PELLEAS Adolescent Sample

**DOI:** 10.3390/ijerph17010003

**Published:** 2019-12-18

**Authors:** Olivier Phan, Constance Prieur, Céline Bonnaire, Ivana Obradovic

**Affiliations:** 1 Clinique Dupré, Fondation Santé des Etudiants de France, 75014 Paris, France; olivier.phan@fsef.net; 2 Centre Pierre Nicole, Consultation Jeunes Consommateurs, 75005 Paris, France; celine.bonnaire@croix-rouge.fr; 3 LPPS Université de Paris, 92774 Boulogne-Billancourt, France; Ivana.Obradovic@ofdt.fr; 4 INSERM UMR-S 1178, Université Paris-Sud, 75014 Paris, France; 5 Observatoire Français des Drogues et des Toxicomanies, CEDEX 7, 75700 Paris, France

**Keywords:** Internet Gaming Disorder, adolescents, satisfaction in life, depression, parental support, economic conditions, gender

## Abstract

Among adolescents, heavy video game use and socializing online may be valued socially by peers, depending on gender and age, which can increase life satisfaction. However, heavy video gaming may also be linked to symptoms of Internet Gaming Disorder, which can decrease life satisfaction. Overall, when symptoms of Internet Gaming Disorder are present, do subjects experience decreased or increased life satisfaction, all other things being equal? The aim of this study was to explore the association between Internet Gaming Disorder symptoms and life satisfaction, while controlling for gender, age, and other conditions that may impact life satisfaction. More than 2000 adolescents filled out an anonymous questionnaire at school, and 43 patients in a care center filled out the same questionnaire. Sociodemographic characteristics, family life conditions, use of screens (videos, video games, and social networks), mental health screenings, and a life satisfaction measure were collected. Distribution of participants’ characteristics was provided, and stratified multivariate analyses by young male, older male, young female, and older female school populations were carried out. Results suggested that Internet Gaming Disorder symptoms had similar prevalence before and after the age of 15 in males (21% vs. 19%) and in females (6% vs. 7%) respectively and was significantly associated with decreased life satisfaction in older males, even after adjusting for parental support, depression, and economic conditions. Associations between symptoms of Internet Gaming Disorder and life satisfaction may be different depending on adolescent gender and age group.

## 1. Introduction

### 1.1. Context and Problem

#### 1.1.1. Context

Internet Gaming Disorder was included in the fifth edition of the *Diagnostic and Statistical Manual of Mental Disorders* (DSM-5) as a condition requiring further study. Because playing video games is a highly valuated and time-consuming activity for adolescents, mental health professionals have acquired an interest in Internet Gaming Disorder (IGD)’s effects on well-being. Research has demonstrated that adolescents’ quality of life would decrease if Internet access was limited [1], that problematic internet use correlates negatively and significantly with health-related quality of life [2], and that Chinese adolescents with problematic Internet use (PIU) have lower scores on total and all dimensions of life satisfaction measures [3] and lower well-being [4] than non-PIU adolescents. A study of elementary school children showed that children with pathological and maladaptive Internet use exhibited more severe depression and lower health-related quality of life [5]. Some authors have demonstrated that adolescents in outpatient care had increased quality of life [6].

#### 1.1.2. Problem

Is IGD itself causing decreased well-being or is it the comorbidities and life conditions associated with IGD that cause decreased well-being? To our knowledge, this question remains unanswered.

### 1.2. Literature Review

Prevalence of IGD varies across studies partly because it depends on the scale used by research teams. A literature review conducted by Mihara and Higuchi found a prevalence of IGD ranging from 0.7% to 27.5%, depending on samples. Prevalence in males was higher than in females and tended to be higher in younger participants. Prevalence rates found in different studies also varied slightly across countries. Factors found to be associated with Internet Gaming Disorder are game characteristics, demographic variables, family socio-economic level, and school functioning outcomes. Factors also include personality traits, psychiatric comorbidities, social relationships, and health conditions [7]. Some authors have tried to determine the relative impact of these factors on IGD with step-wise regressions. Explained variance of addictive use of technologies was 11% to 12% with demographic factors, and 7% to 15% with mental health factors [8]. A systematic review on the association between IGD and mental disorders found correlations of 92% between IGD and anxiety, 89% between IGD and depression, and 85% between IGD and attention deficit hyperactivity disorder [9]. In Canada, 5820 patients in a treatment center for Internet Gaming Disorder were screened. Logistic regression analyses showed that male gender, age, extreme shyness, internalized symptoms such as symptoms of depression and anxiety, and externalized symptoms such as hyperactivity and behavioral disorders were all significantly associated with IGD [10]. In a longitudinal study, it was demonstrated that gaming frequency at baseline predicted an increase in internalizing problems one year later [11]. The relationship between a deficit in resilience and occurrence of IGD may be mediated by relational difficulties with peers [12]. Three waves of longitudinal European studies named “Saving and Empowering Young Lives in Europe” showed that early symptoms of IGD and emotional problems were predictors of developing and IGD later [13]. Rokkum and Gentile wrote that “IGD is likely best conceptualized as a disorder that can be comorbid or multimorbid” [14]. There are many types of video games, and there are many reasons to play video games. Gaming to avoid negative feelings and gaming for fun or for social reasons should be distinguished. Indeed, “weekday online gaming for more than five hours a day, in combination with an avoidance motivation, was associated with an increased probability of depressive symptoms”, whereas “the probability of ill health decreased when gaming was for fun or had social motives” [15]. In their study, Li and colleagues found that “(a) depression mediated the relationship between Actual–Ideal Self Discrepancies and escapism, and (b) escapism mediated the relationship between depression and IGD” [16]. In a study that performed latent class analyses on a sample of 9733 adolescents interviewed at school, Colder Carras and colleagues identified two types of heavy gaming that differed in their quantity of online social interaction. Classes with more online social interaction reported fewer problematic gaming symptoms than those with less online social interaction [17]. In summary, IGD is a complex and unclear entity, related to many factors, occurring mainly among adolescents with low psychosocial skills and social support. “IGD may aggravate existing deficits and vice versa; poor social relations will motivate gaming, and spending increased time gaming will aggravate poor relationships, thus further reinforcing IGD” [18]. It is unclear whether IGD alone impacts well-being, or if the suffering comes from psychosocial characteristics associated. In the present study, psychosocial difficulties, identified with standardized tools, and elements of health, family, school, economic, and cultural conditions were collected in a sample of adolescents at school, and in a sample of patients in care centers. Then, controlling for several factors, the effect of symptoms of Internet Gaming Disorder alone on life satisfaction was studied.

### 1.3. Contribution

Few studies on the association between subjective well-being of adolescents and IGD have considered such a large amount of characteristics of adolescents’ lives. To our knowledge, no study has collected standardized data from a school-adolescents sample and a patient sample at the same time. In this study, adolescent depression, social phobia, and symptoms of IGD were measured using validated self-report measures.

### 1.4. Aim of the Study

The main objective of the study was to determine whether suffering from IGD symptoms was related to decreased life satisfaction in the adolescent sample (recruited in the Programme d’étude sur les liens et l’impact des écrans chez les adolescents scolarisés: Study program concerning the links and impact of screens in school adolescents (PELLEAS) study), while controlling for life conditions and mental health conditions, and to investigate how this association differed by gender and age.

## 2. Methods

### 2.1. Participants

**Figure 1:**
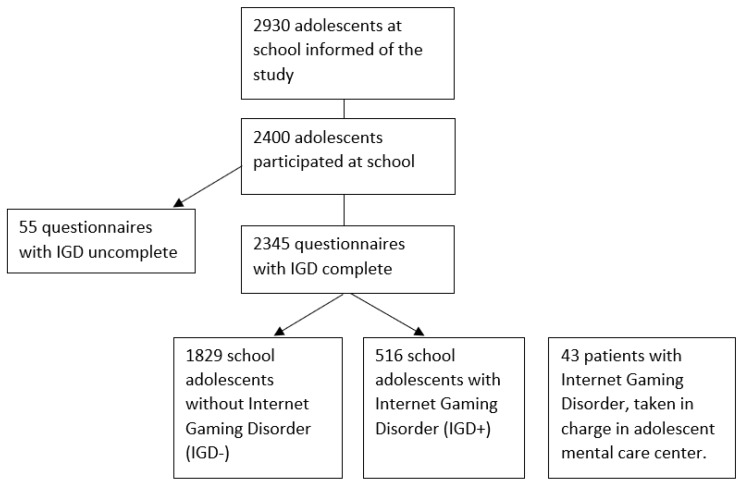
Flow-chart.

During the PELLEAS study, adolescents from schools were included first, then adolescent patients from care centers. Samples were recruited from November 2013 to December 2014. Adolescents were recruited from the general population, in 20 schools in Paris and the suburbs, and amounted to a total of 2400 adolescents. Ages ranged from 12 to 20. The 20 schools chosen for recruitment purposes were selected so that the sample of school children would have the same socioeconomic characteristics as the population from the addiction centers. Indeed, the French Observatory for Drug and Addictive Behaviors (OFDT) recommends recruitment of samples that are similar in terms of geographic criteria, success rates at the Baccalauréat (end of high school exam), state or private management, and general scope of the teaching programs, in order to minimize socioeconomic bias. The general school population sample was screened with the game addiction scale (GAS), a validated pathological gaming self-report questionnaire [19]. According to their results on this scale, the school participants were divided into two different subgroups: adolescents with symptoms of IGD (school adolescents who tested above the threshold on the GAS, that is who had at least four positive items out of seven) and adolescents without symptoms of IGD. The IGD patients were recruited from two specialized centers for adolescents with addictive behaviors in Paris, France (Centre Croix Rouge Pierre Nicole) and in a Parisian suburb (Clinique Dupré, Fondation Santé des Etudiants de France, Sceaux, France) (see Figure 1). When a skilled clinician diagnosed IGD after evaluation, the patient was invited to participate in the study. These IGD patients then filled the GAS, but some had a score under the threshold of 4/7. They were still included in the study because most of the adolescents from the clinic consultations denied their gaming use in self-assessment measures, because they were forced by their parents to come to the consultation. Adolescents were in the treatment center for “IGD symptoms”, whereas the school adolescents filled a questionnaire about their gaming habits. The treatment center participants had already heard the terms “IGD symptoms”. We think that in this situation a denial is more likely to occur in reaction to the parents’ decision to call a therapist. Adults’ perception of their misuse and the adolescents’ own perception were different. Clinical assessment and self-questionnaires yielded different results, especially on video game use. This explains the discrepancies in GAS results.

### 2.2. Procedure

All participants filled the same questionnaire. The adolescent population in treatment centers filled the same questionnaire as the adolescent population in schools. In addition, the adolescents in treatment centers were interviewed by skilled therapist, with the Mini-International Neuropsychiatric Interview (MINI).

Participants completed the questionnaire in about 40 min, and provided sociodemographic data, information about their habits concerning use of media, and about their support from family and friends. Most of the school participants (89%) reported feeling comfortable with the questionnaire. The adolescent treatment center population and the school population were both recruited in Paris and the suburbs. However, matching failed: the two populations were not considered similar in terms of gender, age, body mass index, limitations of access to screens, academic results, and parental marital status. Participants answered a variety of clinical scales described below.

### 2.3. Measures

#### 2.3.1. Life Satisfaction Scale

At the end of the questionnaire, individuals were asked to fill a visual analogic scale of life satisfaction, the Cantril’s self-anchoring ladder rating of life. From 0 (the worst life possible) to 10 (the best life possible), the respondents were invited to choose one level of the scale to assess their current life perception. “Given its subjective nature, one of the major ways subjective well-being has been measured is through self-report rating scales, which privileges personal evaluations and experiences. These self-report measures have good reliability” [20] The measure is easy and straight-forward. “Quality of life can be suitably expressed if patients are simply invited to give it their own rating on a global scale” [21]. The Cantril’s self-anchoring ladder rating of life was used as soon as 1965 to compare subjective well-being in different countries, with responses varying from 1 = worst possible life to 10 = best possible life [20]. In our sample 8% of the data were missing for the life satisfaction scale. Unfortunately, individuals with symptoms of IGD (IGD+) and depressed individuals were over-represented in the missing data. First, we excluded it from the analysis. Then, as a check for robustness, in a second stage, we assumed that subjects for whom data were missing had a mean quality of life score of 7.32 for the multivariate analysis. 7.32 was chosen because it was the mean quality of life value in the school sample.

#### 2.3.2. Gaming Use

Each participant filled the short version of the game addiction scale for adolescents (GAS) developed by the Lemmens and Valkenburg team to evaluate gaming disorder. Each item represents one of the following criteria based on the definition of behavioral addiction: salience, tolerance, mood modification, withdrawal, relapse, conflict, and problems. Responses were scored on a five-point Likert scale ranging from 1 (never) to 5 (very often). The seven-item GAS shows good internal consistency and good concurrent validity in a French adolescent population. This is also indicated by the consistent correlations with usage, loneliness, life satisfaction, social competence, and aggression [22]. This scale specifically addresses the developmental characteristics of adolescents and young adults. For example, some items refer to individuals’ homework or parents (which would not be suitable items for children or adults). Thus, this tool is adapted to the population studied. In 2013, the inclusion and definition of IGD in the DSM was based partly on the definition of substance-related disorders and mostly on the definition of non-substance related disorders. Thus, the GAS and the DSM criteria for IGD are very similar but not equivalent. Indeed, the GAS was developed years before the appearance of the DSM-5, therefore it covers only seven out of nine criteria. As recommended by the authors, four validated items (a validated item means a response > 2 (sometimes or more)) correspond to problematic use of video games. This cut-off point is in line with the polythetic format applied in the Diagnostic and Statistical Manual of Mental Disorders-5th edition (DSM-V) (American Psychiatric Association) [19], i.e., at least half of the criteria indicated that the subject’s video game use is problematic. In the study population, the validity of the scale was assessed. There was neither a ceiling effect nor a floor effect in the distribution of answers to the seven items. The unidimensional nature of the scale was confirmed which allowed the calculation of a global score with the addition of the score of each item. Cronbach’s alpha was 0.84. Some data were missing for the questions on IGD symptoms in the questionnaires. We excluded them from the analyses because they accounted for less than 3% of the data. We observed that data was missing more often when the individuals were males (60%) with higher depression scores and aged 14 or more.

#### 2.3.3. Adolescent Depression Rating Scale

The 10-item self-report version of the adolescent depression rating scale (ADRS) was used in this study. It is known to discriminate adolescents with and without depression better than the Hamilton depressive rating scale or the Beck depression inventory (BDI-13) [23]. Validated in French, and specific to the adolescent population, it had good internal consistency in this sample, with a Cronbach’s alpha coefficient >0.70. It was used to screen depression among respondents. Missing values represented less than 5% of the sample for the depression score, so we excluded them from the analysis.

#### 2.3.4. The Liebowitz Social Anxiety Scale

The Liebowitz social anxiety scale (LSAS) is a well-validated scale used to assess the dimensional severity of social anxiety disorder symptoms. The LSAS is a 24-item scale that measures fear and avoidance of social situations over the past week. It consists of 11 items relating to social interaction and 13 items related to public performance. Each item is rated on two four-point Likert-type scales. The first rating is a measure of fear/anxiety and ranges from 0 (none) to 3 (severe). The second rating is a measure of avoidance and ranges from 0 (never) to 3 (usually; 68%–100%). A total score is calculated by summing up all of the fear and avoidance ratings. The LSAS has good psychometric properties [24].

#### 2.3.5. Age and Gender

Age in the sample is normally distributed, with a mean at 14.81 (0.028) in the school sample, which is approximately the age of leaving school institution “college” (age 10–15) for “lycée” (age 14–18), in France. In “Adolescence and Psychopathology” (8th ed. Elsevier Masson), D. Marcelli and A. Braconnier write that age and gender are parameters which have a huge influence on incidence and prevalence of mental health troubles in adolescence. With age, the difference of prevalence for issues between females and males increase. For this reason, it was more appropriate to run analyses separately for males and females, and for younger and older participants.

### 2.4. Analyses

First, we measured the frequency of different characteristics among school adolescents who were IGD+, school adolescents who didn’t have symptoms of IGD (IGD−), and IGD patients. We measured sociodemographic characteristics such as age, sex, economic living conditions, and sociocultural background measured by a proxy (number of books at home). Health characteristics, regarding height, weight, body image, sleep, depression, and social phobia were computed. Data on family habits was collected through questions about dinner circumstances, parental support, professional situation of mother and father, and people living at home with the respondent. Data concerning school results, relationships with friends, and video games features was also collected. We did not perform chi-squared tests for comparison, because the group of schoolers and the patients were not the same age (14.83 (IC95% 14.73–14.93) vs. 15.74 (IC95% 14.98–16.50)). Results are presented in Table 1, Table 2, Table 3, Table 4, Table 5, Table 6, Table 7 and Table 8. Secondly, we analyzed the association between symptoms of IGD and quality of life in the school population. Multivariate analyses were performed in each gender group and in each age class. There were 480 observations in females under the age of 15 (of which 30 (6%) were IGD+), 701 observations in females aged 15 and more (of which 50 (7%) were IGD+), 456 observations in males under the age of 15 (of which 98 (21%) were IGD+), and 687 observations in males aged 15 and more (of which 130 (19%) were IGD+). With a linear regression model, we predicted quality of life with symptoms of IGD, controlling for sociodemographic variables, health variables, and life conditions. All things being equal, we were able to determine the association between symptoms of IGD and quality of life. Results are shown in Table 9 and Table 10.

## 3. Results

Male adolescents scored above the threshold for symptoms of IGD more frequently than girls (20% vs. 7%, *p* < 0.000). From the medical point of view, IGD+ subjects weighed less than the optimal weight more frequently than IGD− subjects. They also reported going to bed later. Quality of life was lower and depression and social phobia symptoms were more frequent among IGD+ and patient samples compared to IGD− subjects. Social phobia was very high in male and female populations, with a greater intensity among IGD+ participants. Among female participants, quality of life was very low, and depression was frequent, when symptoms of IGD were present. In the patient group, there were only male participants. They were older and had higher prevalence of depression and anxiety than the adolescents from the two school samples. They also used tobacco, alcohol, and cannabis more frequently than the adolescents from the two school sample. See Table 1 and Table 2.

Patients reported living with both parents less often than schoolers. In the whole sample, fathers and mothers were less often in employment when symptoms of IGD were present. IGD+ subjects had dinner alone or in front of screens frequently, and reported having dinner less often with family (one adult present) and less often at a fixed time than IGD- subjects. Socio-economic conditions were less good for IGD+ subjects, male or female, and less parental and amical support were reported (Table 3 and Table 4).

Marks at school were slightly different across samples. Reports of stress at school was subject to variation inside samples. The desire to pursue studies after high school was lower in IGD+ school and patient samples, whereas more adolescents in IGD+ and patient samples had already repeated a year at school. Number of books at home, which was a proxy for the sociocultural level of the family, suggested that symptoms of IGD were more frequent in adolescents from families with a low sociocultural level. However, a majority of patients reported a number of books similar to the IGD- population. We do not know if this observation corresponds to the fact that the treatment centers were in high-income areas, or if access to care was better for families with a high sociocultural level. Number of real friends in real life was lower in IGD+ school and patient samples, whereas number of real friends in virtual life was higher mainly in the IGD+ sample. (Table 5 and Table 6).

School adolescents with symptoms of IGD and patients reported more frequently logging on late, falling asleep in front of screens, skipping meals to stay connected, and refusing to go out to stay connected. Patients showed more intensity in these behaviors than IGD+ subjects, suggesting a gradient in the severity of the problem. Parents and teachers seemed to react to these behaviors, as access to screens was systematically forbidden more often, and telephones were confiscated in class more often in the IGD+ sample than in the IGD− sample. Time spent in front of the TV and on the internet, and compulsion in the use of social networking was higher in IGD+ subjects than in IGD− subjects, suggesting a high use of screens in general. Use of Internet for more than 2 h per day was extremely high in female adolescents. Playing simulation games was high in schoolers, and relatively low in patients. This may have been related to the fact that simulation games are easily played with others in the same room, making video games a part of social life, and patients may have lacked socialization with peers; playing alone and playing online were more frequent in IGD− subjects and in patients. (Table 7 and Table 8)

### Associations between IGD and Quality of Life

A multivariate analysis was conducted to investigate the links between quality of life and symptoms of IGD. We ran several models of linear regression, to predict quality of life with status of IGD symptoms (present or absent) for separate gender and age classes (under 15 and 15 and over), controlling for factors such as individual factors, the parental support score, socioeconomic conditions, and depression. In male adolescents, incidence of symptoms of IGD was similar before and after the age of 15. Symptoms of IGD were significantly associated with decreased quality of life in males aged 15 and more, even when adjusting for depression, economic conditions, and parental support, and with a smaller effect in males aged under 15. In female adolescents, incidence of symptoms of IGD was similar before and after the age of 15. It was not significantly associated with decreased quality of life in females under and over 15, when adjusting for depression, age, economic conditions, and parental support. (Table 9).

When we replaced the missing data for quality of life with the mean quality of life in the sample, the value of which was 7.32, we obtained similar results (Table 10).

## 4. Discussion

### 4.1. Contributions

Few studies had shown the effect of symptoms of IGD on quality of life, taking into account main determinants of life satisfaction among adolescents.

### 4.2. Principal Results

This study showed that when taking into account variables such as sociodemographic, economic, health, and family conditions variables, the negative association between symptoms of IGD and quality of life was still significant in male adolescents. It showed that male and female adolescents differed in terms of associations: symptoms of IGD were significatively associated with decreased quality of life when male adolescents were 15 and older, whereas no significant impact was found in female adolescents. The female adolescent sample may have been too small for a significant impact to be shown. Results suggest that quality of life may be independently affected by symptoms of IGD, when familial conditions (parental support and socio-economic conditions) and depression are accounted for. The strength of the association may have depended on the period of life for the adolescents, and this period of life differed between male and female adolescents. The description of the sample provided with some interesting insights: for instance, adolescents went to bed late, even more when they were IGD+, and presence of screens inside family life was more prominent in IGD+ group. A few females were IGD+, but IGD+ females presented lower satisfaction in life and more frequent depression symptoms than IGD+ males.

### 4.3. Limitations

This study may be subject to moderate selection bias related to the written nature of the interviews, meaning only patients who were physically present completed them, as opposed to online questionnaire. Variations in completion may also be related to the IGD symptoms. No school adolescent present in class the day of the interviewer’s interventions refused to participate in the study. However, questionnaires were filled out in heterogeneous ways: some answers were missing, especially at the end of the questionnaire, or when the question addressed an issue with potential emotional entanglement for the adolescent, or on the contrary, an issue which did not seem interesting to the adolescent. Additionally, some adolescents suffering from symptoms of IGD may have not attended classes and thus potentially may have not participated in the study. We do not have any information on the reason for students not participating (absence from school or refusal to participate). This could result in underestimation of the prevalence of IGD symptoms, and in underestimation of the association between symptoms of IGD and decreased quality of life.

The prevalence of IGD symptoms in the school population could be inflated due to the GAS measure, adapted from substance abuse scales. Even if the scale was validated in the population, and unidimensional, specificity of the measure could not be assessed. This raises the concern of how appropriate the instrument used (the GAS) is for classifying gaming disorder, and whether it overestimates prevalence. The GAS was developed years before the appearance of the DSM-5, therefore it covers only seven out of nine criteria. The use of a diagnostic instrument such as the Internet Gaming Disorder scale–9-short-form [25] in future research may be more appropriate. The Internet-Gaming-Disorder scale, proposed by the same research team that proposed the GAS in 2015, seems to have better metrological properties. Unfortunately, students completed the GAS because the data collection took place in 2013–2014.

Additionally, while our data was collected six years ago, we think that the problem highlighted in this study is still current and accurate. Indeed, in our clinical practice, the number of admissions for IGD is ever increasing.

Patients in care centers and adolescents at school were not comparable in terms of sociodemographic characteristics (age, gender). The group in treatment were mostly boys, which is the case in most consultations, the prevalence of IGD among males in the general population tending to be higher than the prevalence of IGD among girls. During our inclusion period, no girls were present in the care centers. This result is in accordance with studies on IGD showing higher prevalence of IGD symptoms among males [26,27]). The age was also higher among the patient group. Indeed, it generally takes time before parents realize that video gaming is a problem for their child. Generally, they bought the videogame for their child and sometimes used it as “a babysitter” (keeping their child busy). Nevertheless, video-gaming does not always lead to negative outcomes. The aim of this study was to measure impact of IGD symptoms on quality of life. A homogenous sample was needed, thus the patient sample were not included in the multivariate analysis because patients and school adolescents showed differences in characteristics (sociodemographic and life conditions). Statistical weighting was not used. Finally, this study was carried out in an area within 30 km of Paris, in a mostly urban area. Results cannot be generalized to rural areas or other regions.

### 4.4. Perspectives

Some studies suggest that excessive video gaming could be used as a way to escape from negative feelings. Our study suggests that IGD symptoms are associated with decreased quality of life in male adolescents, all other things being equal. Future studies could investigate excessive video gaming, to determine whether videogames used specifically as a refuge by adolescents might lead to IGD symptoms and distress.

It was shown elsewhere that low resilience was associated with a higher risk of IGD symptoms [28]. Improving resilience, reducing adverse life conditions through psychosocial skills training, or improving familial support could contribute to preventing feelings of loneliness and pathological involvement in video games. Some interventions have already been developed with a focus on parent–child interactions and evaluated with RCT [29]. Still, further understanding of IGD symptoms among female adolescents is needed, as the pattern of association with quality of life may differ compared to that found in male adolescents.

## Figures and Tables

**Table 1:** Socio-demographic and health variables: males.

Variables	IGD−	IGD+	Patients
Age	14.83 (0.05)	14.86 (0.09)	15.74 (0.38)
BMI	20.07 (0.11)	20.48 (0.38)	21.09 (0.84)
-18	126 (15%)	34 (16%)	4 (10%)
18–25	658 (76%)	157 (72%)	29 (73%)
25–30	73 (8%)	20 (9%)	5 (13%)
+30	9 (1%)	6 (3%)	2 (5.00%)
Body image			
-Far too skinny	11 (1%)	5 (2%)	6 (15%)
-Too skinny	159 (18%)	52 (23%)	4 (10%)
-Appropriate weight	563 (62%)	122 (54%)	21 (51%)
-Too fat	155 (17%)	39 (17%)	8 (20%)
-Far too fat	15 (2%)	9 (4%)	2 (5%)
Binge drinking last month			
-Never	646 (73%)	163 (77%)	28 (68%)
-Once or twice	147 (17%)	25 (12%)	8 (20%)
-3 to 5 times	57 (6%)	13 (6%)	3 (7%)
-More than 6 times	38 (4%)	13 (6%)	2 (5%)
Smoking weekly	146 (17%)	34 (15%)	11 (28%)
Drugs intake at least once			
-Cannabis	264 (29%)	63 (28%)	18 (42%)
-Others	48 (5%)	12 (6%)	4 (10%)
Time gone to bed during week			
-Before 10.30 p.m.	328 (36%)	71 (32%)	12 (31%)
-10.30 p.m. to midnight	444 (49%)	93 (42%)	15 (38%)
-After midnight	130 (14%)	56 (25%)	12 (31%)
Time gone to bed during week-ends			
-Before 10.30 p.m.	75 (8%)	10 (5%)	3 (7%)
-10.30 p.m. to midnight	338 (38%)	56 (26%)	12 (29%)
-After midnight	488 (54%)	153 (70%)	26 (63%)
Severe depression	83 (9%)	40 (18%)	12 (28%)
Social phobia	163 (18%)	52 (23%)	4 (9%)
Quality of life			
-5/10	91 (11%)	39 (19%)	20 (53%)
6–8/10	536 (63%)	134 (65%)	16 (42%)
+8/10	219 (26%)	32 (16%)	2 (5%)
Observations	915	228	43

**Table 2:** Socio-demographic and health variables: females.

Variables	IGD−	IGD+
Age	14.79 (0.04)	14.78 (0.14)
BMI	19.68 (0.09)	20.25 (0.38)
-18	161 (15%)	10 (14%)
18–25	807 (77%)	56 (78%)
25–30	73 (7%)	4 (6%)
+30	5 (0%)	2 (3%)
Body image		
-Far too skinny	15 (1%)	0 (0%)
-Too skinny	83 (8%)	6 (8%)
-Appropriate weight	520 (48%)	41 (56%)
-Too fat	379 (35%)	17 (23%)
-Far too fat	90 (8%)	9 (12%)
Binge drinking last month		
-Never	862 (80%)	57 (80%)
-Once or twice	136 (13%)	8 (11%)
-3 to 5 times	62 (6%)	4 (6%)
-More than 6 times	18 (2%)	2 (3%)
Smoking weekly	188 (17%)	14 (18%)
Drugs intake at least once		
-Cannabis	266 (24%)	20 (26%)
-Others	39 (4%)	7 (10%)
Time gone to bed during week		
-Before 10.30 p.m.	437 (40%)	34 (44%)
-10.30 p.m. to midnight	507 (47%)	29 (38%)
-After midnight	144 (13%)	14 (18%)
Time gone to bed during week-ends		
-Before 10.30 p.m.	103 (10%)	8 (11%)
-10.30 p.m. to midnight	428 (39%)	23 (30%)
-After midnight	553 (51%)	45 (59%)
Severe depression	163 (15%)	29 (36 %)
Social phobia	150 (14%)	24 (30%)
Quality of life		
-5/10	169 (17%)	21 (32%)
6–8/10	621 (61%)	35 (53%)
+8/10	231 (23%)	10 (15%)
Observation	1101	80

**Table 3:** Home and family life variables: males.

Variables	IGD−	IGD+	Patients
Lives with mother	846 (92%)	207 (91%)	31 (72%)
Lives with father	674 (74%)	159 (70%)	22 (51%)
Lives with mother and father	639 (70%)	151 (66%)	17 (40%)
Lives with sibling(s)	685 (75%)	176 (77%)	19 (44%)
Lives with gdmother(s)/gdfather(s)	52 (6%)	14 (6%)	4 (9%)
Lives with m. and f. and sibling(s)	532 (58%)	131 (57%)	11 (26%)
Father situation			
-At work	748 (82%)	186 (82%)	27 (63%)
-Unemployed, looking for a job	31 (3%)	12 (5%)	2 (5%)
-Other	125 (14%)	33 (14%)	13 (30%)
Mother situation			
-At work	721 (79%)	176 (77%)	35 (81%)
-Unemployed, looking for a job	47 (5%)	15 (7%)	2 (5%)
-Other	133 (15%)	44 (19%)	7 (16%)
Dinner alone	87 (10%)	21 (9%)	11 (26%)
Dinner with siblings	77 (9%)	28 (13%)	1 (2%)
Dinner with family	681 (77%)	161 (73%)	26 (62%)
Dinner at friends	11 (1%)	6 (3%)	2 (5%)
No dinner	8 (1%)	2 (1%)	0
Dinner with screen (TV/computer)	163 (19%)	64 (29%)	12 (32%)
Dinner at fixed time	542 (61%)	112 (51%)	23 (55%)
Time mother returns home			
Before 6 p.m.	376 (45%)	88 (42%)	10 (27%)
Between 6 p.m. and 8 p.m.	403 (48%)	105 (50%)	25 (68%)
After 8 p.m.	59 (7%)	15 (7%)	2 (5%)
Time father returns home			
Before 6 p.m.	233 (31%)	52 (27%)	7 (25.00%)
Between 6 p.m. and 8 p.m.	407 (53%)	102 (53%)	19 (68%)
After 8 p.m.	122 (16%)	37 (19%)	2 (7%)
Family economic conditions			
-Average/worse than other families	340 (37%)	96 (42%)	23 (53%)
-Better than other families	575 (63%)	132 (58%)	20 (47%)
Pocket money monthly/weekly	546 (62%)	129 (60%)	23 (53%)
Holiday departures +5/yr	285 (32%)	65 (30%)	11 (26%)
1.Very often clear rules	209 (24%)	45 (21%)	6 (15%)
2.Very often parents know where I am the evening	452 (52%)	93 (43%)	15 (39%)
3.Very often parents know with whom I am	514 (58%)	100 (50%)	19 (49%)
4.Very often moral support from parents	471 (53%)	92 (44%)	11 (26%)
5.Very often easy to speak with parents	459 (52%)	93 (44%)	15 (36%)
Parental support score (sum 1–5.)			
16/20 and more	494 (54%)	105 (46%)	15 (35%)
very often moral support from friends	444 (50%)	104 (49%)	20 (48%)
Observations	915	228	43

**Table 4:** Home variables and Family Life: females.

Variables	IGD−	IGD+
Lives with mother	1031 (94%)	69 (86%)
Lives with father	744 (68%)	44 (55%)
Lives with mother and father	709 (64%)	42 (53%)
Lives with sibling(s)	844 (77%)	57 (71%)
Lives with gdmother(s)/gdfather(s)	51 (5%)	5 (6%)
Lives with m. and f. and sibling(s)	596 (54%)	36 (45%)
Father situation		
-At work	871 (79%)	58 (73%)
-Unemployed, looking for a job	47 (4%)	2 (3%)
-Other	156 (14%)	20 (25%)
Mother situation		
-At work	872 (79%)	56 (70%)
-Unemployed, looking for a job	77 (7%)	5 (6%)
-Other	160 (15%)	16 (25%)
Dinner alone	96 (9%)	7 (10%)
Dinner with siblings	103 (10%)	8 (11%)
Dinner with family	828 (77%)	51 (72%)
Dinner at friends	8 (1%)	1 (1%)
No dinner	22 (2%)	0
Dinner with screen (TV/computer)	241 (22%)	25 (34%)
Dinner at fixed time	617 (57%)	40 (52%)
Time mother returns home		
Before 6 p.m.	408 (39%)	35 (49%)
Between 6 p.m. and 8p.m.	549 (53%)	27 (38%)
After 8 p.m.	82 (8%)	9 (13%)
Time father returns home		
Before 6 p.m.	225 (26%)	15 (26%)
Between 6 p.m. and 8 p.m.	479 (54%)	30 (53%)
After 8 p.m.	175 (20%)	12 (21%)
Family economic conditions		
-Average/worse than other families	404 (37%)	42 (53%)
-Better than other families	697 (63%)	38 (48%)
Pocket money monthly/weekly	660 (61%)	39 (52%)
Holiday departures +5/yr	388 (36%)	17 (22%)
1.Very often clear rules	282 (26%)	22 (31%)
2.Very often parents know where I am the evening	719 (67%)	35 (49%)
3.Very often parents know with whom I am	777 (73%)	38 (54%)
4.Very often moral support from parents	456 (42%)	14 (20%)
5.Very often easy to speak with parents	546 (51%)	21 (30%)
Parental support score (sum 1–5.)		
16/20 and more	645 (59%)	38 (48%)
Very often moral support from friends	757 (70%)	40 (57%)
Observations	1101	80

**Table 5:** School and friendship variables: males.

Variables	IGD−	IGD+	Patients
General average mark			
above 15/20	79 (9%)	16 (7%)	2 (5%)
from 10 to 15/20	707 (80%)	162 (75%)	20 (54%)
Average mark in maths			
above 15/20	164 (18%)	40 (18%)	4 (11%)
from 10 to 15/20	478 (53%)	120 (54%)	18 (50%)
Average mark in french			
above 15/20	67 (7%)	7(3%)	3 (9%)
from 10 to 15/20	598 (67%)	146 (66%)	14 (41%)
Average mark in history			
above 15/20	102 (11%)	22 (10%)	2 (6%)
from 10 to 15/20	659 (74%)	149 (68%)	23 (64%)
Feeling not at all stressed at school	311 (34%)	66 (29 %)	15 (36%)
Feeling very stressed at school	112 (12%)	43 (19%)	12 (29%)
Desire to pursue studies after high school	662 (75%)	143 (66%)	19 (46%)
Has repeated a year	155 (17%)	48 (22%)	15 (38%)
Number of books at home			
From 0 to 10	103 (12%)	33 (15%)	3 (7%)
From 10 to 50	166 (19%)	43 (20%)	8 (19%)
From 50 to 100	161 (18%)	49 (22%)	8 (19%)
More than 100	462 (52%)	93 (43%)	23 (55%)
3+ real friends in real life	577 (64%)	124 (56%)	19 (46%)
3+ real friends in virtual life	190 (21%)	77 (35%)	9(23%)
Observations	915	228	43

**Table 6:** School and friendship variables: females.

Variables	IGD−	IGD+
General average mark		
above 15/20	132 (12%)	6 (8%)
from 10 to 15/20	834 (78%)	55 (77%)
Average mark in maths		
above 15/20	177 (17%)	10 (13%)
from 10 to 15/20	609 (57%)	34 (45%)
Average mark in french		
above 15/20	128 (12%)	8 (11%)
from 10 to 15/20	763 (72%)	50 (67%)
Average mark in history		
above 15/20	149 (14%)	9 (12%)
from 10 to 15/20	749 (70%)	43 (58%)
Feeling not at all stressed at school	209 (19%)	23 (29%)
Feeling very stressed at school	304 (28%)	20 (26%)
Desire to pursue studies after high school	883 (82%)	47 (59%)
Has repeated a year	150 (14%)	19 (25%)
Number of books at home		
From 0 to 10	85 (8%)	9 (12%)
From 10 to 50	228 (21%)	25 (33%)
From 50 to 100	221 (20%)	15 (20%)
More than 100	555 (51%)	26 (35%)
3+ real friends in real life	636 (58%)	42 (54%)
3+ real friends in virtual life	130 (12%)	22 (29%)
Observations	1101	80

**Table 7:** Video game variables: males.

Variables	IGD−	IGD+	Patients
Very often late connecting	144 (16%)	66 (29%)	23 (55%)
Often/very often fell asleep in front of computer/mobile device	53 (6%)	42 (19%)	6 (14%)
Often/very often skips a meal to stay connected	50 (6%)	55 (24%)	15 (37%)
Often/v.often refuses to go out/activity to stay online	27 (3%)	47 (21%)	11 (26%)
Often/v.often forbidden access to screen	75 (8%)	46 (20%)	12 (28%)
Often/v.often got my phone confiscated in class	30 (3%)	16 (7%)	3 (7%)
In front of TV +2 h/day	317 (35%)	123 (54%)	9 (21%)
On the internet +2 h/day	649 (71%)	194 (85%)	38 (88%)
Types of video game played weekly			
-First person shooter and action	596 (68%)	169 (80%)	29 (69%)
-Role playing game	302 (35%)	103 (47%)	25 (61%)
-Adventure	375 (43%)	114 (54%)	16 (39%)
-Real time strategy	261 (30%)	105 (51%)	27 (68%)
-Simulation (football/cars/sports)	546 (62%)	150 (71%)	16 (40%)
-Managing (Sims...)	126 (15%)	47 (23%)	7 (18%)
-Applications (CandyCrush, AngryBirds...)	606 (70%)	165 (78%)	23 (58%)
Very often playing alone	169 (19%)	78 (36%)	18 (44%)
Very often playing online	228 (25%)	120 (56%)	32 (76%)
Often/very often playing with others in the same room	312 (35%)	73 (34%)	1 (2%)
Observations	915	228	43

**Table 8:** Video game variables: females.

Variables	IGD−	IGD+
Very often late connecting	170 (15%)	31(41%)
Often/very often fell asleep in front of computer/mobile device	125 (11%)	17 (23%)
Often/very often skips a meal to stay connected	54 (5%)	21 (18%)
Often/very often refuses to go out/activity to stay online	21 (2%)	10 (13%)
Often/very often forbidden access to screen	52 (5%)	14 (18%)
Often/very often got my phone confiscated in class	21 (2%)	6 (8%)
In front of TV +2 h/day	382 (35%)	50 (63%)
On the internet +2 h/day	763 (69%)	75 (94%)
Types of VG played weekly		
-First person shooter and action	250 (25%)	42 (63%)
-Role-playing game	92 (9%)	20 (30%)
-Adventure	154 (15%)	32 (49%)
-Real time strategy	78 (8%)	21 (33%)
-Simulation (football/cars/sports)	203 (20%)	29 (44%)
-Managing (Sims...)	306 (30%)	37 (56%)
-Applications (CandyCrush, AngryBirds...)	741 (71%)	56 (81%)
Very often playing alone	121 (11%)	23 (33%)
Very often playing online	6 (1%)	7 (10%)
Often/very often playing with others in the same room	167 (15%)	33(48%)
Observations	1101	80

**Table 9:** Linear: Explain quality of life with IGD.

	Males under 15	Males Aged 15+	Females under 15	Females aged 15+
	(1)	(2)	(3)	(4)
IGD	−0.323 *	−0.405 **	−0.390	0.017
	[−0.661, 0.015]	[−0.735, −0.074]	[−0.973, 0.193]	[−0.504, 0.539]
depression	−1.859 ***	−0.577 **	−1.359 ***	−1.290 ***
	[−2.367, −1.351]	[−1.034, −0.120]	[−1.764, −0.955]	[−1.632, −0.949]
economic	0.805 ***	0.533 ***	1.007 ***	0.784 ***
	[0.513, 1.098]	[0.271, 0.795]	[0.701, 1.313]	[0.534, 1.035]
parental support score	0.639 ***	0.610 ***	0.798 ***	0.983 ***
	[0.356, 0.922]	[0.356, 0.864]	[0.509, 1.087]	[0.735, 1.230]
Constant	6.862 ***	6.889 ***	6.430 ***	6.242 ***
	[6.567, 7.158]	[6.634, 7.145]	[6.107, 6.753]	[5.998, 6.486]
Observations	428	623	455	632

95% confidence intervals in brackets; * *p* < 0.10, ** *p* < 0.05, *** *p* < 0.01.

**Table 10:** Linear: Explain quality of life with IGD, after adjustment, imputation of lifescale.

	Males under 15	Males Aged 15+	Females under 15	Females Aged 15+
	(1)	(2)	(3)	(4)
IGD	−0.261	−0.398 ***	−0.375	0.077
	[−0.580, 0.058]	[−0.695, −0.101]	[−0.938, 0.188]	[−0.361, 0.514]
depression	−1.474 ***	−0.432 **	−1.261 ***	−1.058 ***
	[−1.914, −1.034]	[−0.803, −0.060]	[−1.648, −0.873]	[−1.359, −0.758]
economic	0.751 ***	0.471 ***	0.934 ***	0.671 ***
	[0.474, 1.027]	[0.233, 0.709]	[0.644, 1.225]	[0.443, 0.900]
parental support score	0.680 ***	0.570 ***	0.775 ***	0.966 ***
	[0.413, 0.948]	[0.339, 0.801]	[0.498, 1.052]	[0.741, 1.192]
Constant	6.853 ***	6.948 ***	6.483 ***	6.329 ***
	[6.573, 7.134]	[6.718, 7.178]	[6.175, 6.792]	[6.104, 6.553]
Observations	456	687	480	701

95% confidence intervals in brackets; * *p* < 0.10, ** *p* < 0.05, *** *p* < 0.01.
